# Solid–Liquid Equilibrium in Co-Amorphous Systems: Experiment and Prediction

**DOI:** 10.3390/molecules28062492

**Published:** 2023-03-08

**Authors:** Alžběta Zemánková, Fatima Hassouna, Martin Klajmon, Michal Fulem

**Affiliations:** 1 Department of Physical Chemistry, University of Chemistry and Technology, Prague, Technická 5, 166 28 Prague, Czech Republic; zemankol@vscht.cz (A.Z.); klajmonm@vscht.cz (M.K.); 2 Faculty of Chemical Engineering, University of Chemistry and Technology, Prague, Technická 5, 166 28 Prague, Czech Republic

**Keywords:** co-amorphous systems, active pharmaceutical ingredients, phase diagrams, solid–liquid equilibrium, PC-SAFT, COSMO-RS, physical stability, glass transition temperature

## Abstract

In this work, the solid–liquid equilibrium (SLE) of four binary systems combining two active pharmaceutical ingredients (APIs) capable of forming co-amorphous systems (CAMs) was investigated. The binary systems studied were naproxen-indomethacin, naproxen-ibuprofen, naproxen-probucol, and indomethacin-paracetamol. The SLE was experimentally determined by differential scanning calorimetry. The thermograms obtained revealed that all binary mixtures investigated form eutectic systems. Melting of the initial binary crystalline mixtures and subsequent quenching lead to the formation of CAM for all binary systems and most of the compositions studied. The experimentally obtained liquidus and eutectic temperatures were compared to theoretical predictions using the perturbed-chain statistical associating fluid theory (PC-SAFT) equation of state and conductor-like screening model for real solvents (COSMO-RS), as implemented in the Amsterdam Modeling Suite (COSMO-RS-AMS). On the basis of the obtained results, the ability of these models to predict the phase diagrams for the investigated API–API binary systems was evaluated. Furthermore, the glass transition temperature (*T*_g_) of naproxen (NAP), a compound with a high tendency to recrystallize, whose literature values are considerably scattered, was newly determined by measuring and modeling the *T*_g_ values of binary mixtures in which amorphous NAP was stabilized. Based on this analysis, erroneous literature values were identified.

## 1. Introduction

The poor aqueous solubility of a significant number of newly developed active pharmaceutical ingredients (APIs) presents one of the most serious problems in the pharmaceutical industry. Therefore, strategies to enhance the aqueous solubility of APIs, and thus their bioavailability, have been developed intensively in recent years. A promising way to increase bioavailability is API amorphization. The amorphous forms of APIs exhibit a higher dissolution rate and apparent aqueous solubility in comparison with their crystalline counterparts [1,2,3], but are inherently thermodynamically unstable and tend to convert to their original crystalline forms. To stabilize API amorphous forms and improve their dissolution characteristics, formulation strategies based on glass solutions are widely explored. Glass solutions can be classified as polymeric and non-polymeric systems depending on the excipient used [4]. Co-amorphous systems (CAMs), whose phase behavior is studied in this work, belong to non-polymeric glass solutions and are defined as single-phase amorphous mixtures formed by low-molecular-weight components [5]. This work focuses on API–API CAMs, in which one of the APIs acts as an amorphous stabilizer for the second API through various mechanisms such as salt formation, hydrogen bonding, and *π*–*π* interactions [5,6]. Currently, only a small number of API–API CAMs have been reported, as it is challenging to find a combination of APIs from the pharmacologically related group of APIs, which can form stable CAMs.

The type of solid–liquid equilibrium (SLE) phase diagram can be identified based on the thermal analysis performed for a given binary mixture. The thermogram for a binary physical mixture capable of forming a solid compound (i.e., crystalline salt or cocrystal) typically shows an exothermic peak associated with its formation and several endothermic peaks related to metastable eutectic melting, eutectic melting, and solid compound melting [7,8]. For physical mixtures that form a eutectic system without the formation of a solid compound, only two endothermic events corresponding to eutectic melting and dissolution of one of the crystals in the equilibrium melt are recorded in the thermograms. The top of the latter peak presents a good approximation for evaluation of liquidus temperature (*T*_L_) [9].

Recently, Kissi et al. [10] studied the physical stability of three CAMs and its relationship to binary phase diagrams. CAMs corresponding to the eutectic composition were found to exhibit the highest physical stability among the compositions investigated. The same finding for CAMs consisting of naproxen (NAP) and indomethacin (IND) was reported by Beyer et al. [11]. The enhanced physical stability of certain CAMs may also be due to the formation of a solid compound [12], whose formation can be identified from the phase diagram. Therefore, phase diagrams for mixtures with the potential to form CAMs can provide valuable insight into the mechanisms of their stabilization and the identification of optimal conditions for their preparation. For instance, on the basis of the phase diagram, the optimal composition corresponding to the eutectic mixture or the stoichiometry of a solid compound can be identified. When melting-based approaches are used, the phase diagrams can identify the optimal temperature for CAM preparation (above *T*_L_), which can be significantly lowered compared with the melting temperature of pure APIs, thus preventing their thermal decomposition.

NAP exhibits poor glass-forming ability, i.e., a high tendency to recrystallize, and is classified as Class 1 according to the classification system established by Baird et al. [13]. As a result of its high recrystallization tendency, the preparation of stable amorphous solid formulations containing NAP is challenging. In this work, the phase behavior of three binary NAP systems with APIs with good glass-forming ability (indomethacin (IND), ibuprofen (IBU), and probucol (PRO)) belonging to Class 3 was investigated. Based on the measurement and modeling of the glass transition temperature (*T*_g_) of binary amorphous mixtures in which NAP was stabilized in the amorphous state, its *T*_g_ was determined and used to reconcile the values in the literature [2,14,15,16], showing significant discrepancies. The fourth binary system investigated in this work was IND-paracetamol (PAR), as the combination of these two APIs proved to have synergetic effects in the treatment of active rheumatoid arthritis [17] and their co-amorphous formulation exhibited improved physical stability, dissolution, and supersaturation of IND [18].

Phase diagrams for the four binary systems, namely, NAP-IND, NAP-IBU, NAP-PRO, and IND-PAR, were experimentally determined by differential scanning calorimetry (DSC) and compared to theoretical predictions using (i) the perturbed-chain statistical associating fluid theory (PC-SAFT) equation of state (EOS) [19,20] and (ii) the conductor-like screening model for real solvent (COSMO-RS) as implemented in the Amsterdam Modeling Suite (COSMO-RS-AMS) [21,22]. These computational models represent two different approaches to modeling thermodynamic properties. The PC-SAFT is one of the most widely used advanced EOS derived from the statistical associating fluid theory [23], while COSMO-RS-AMS is a quantum-chemistry-based model. Based on the obtained results, the performance of these models was evaluated as part of our long-term research activities related to the rational design of drug delivery systems with the help of theoretical modeling [24,25].

## 2. Results and Discussions

### 2.1. Thermodynamic Fusion Properties and Glass Transition Temperatures of Pure APIs

First, the polymorphic forms of the APIs studied were identified by X-ray powder diffraction (see Figure S1 in the Supplementary Materials) and comparison to the Cambridge structural database (CSD) [26]. The melting temperatures *T*_m_ and the enthalpies of fusion Δ_fus_*H* of given polymorphs and the glass transition temperatures *T*_g_ obtained in this work are listed in Table 1. The reported values for *T*_m_, Δ_fus_*H*, and *T*_g_ were measured using the same conditions as the data for binary phase diagrams to ensure the consistency between the two datasets. The fusion thermodynamic properties for IND, IBU, and NAP, as well as *T*_g_ values for IND and IBU, are in close agreement with those reviewed and summarized by Štejfa et al. [27]. The *T*_g_ value of NAP was determined in this work based by extrapolation from the measured *T*_g_ values of binary mixtures with NAP (for details and comparison to the literature values, see Section 3.3.4). In the case of PAR and PRO, *T*_m_ and Δ_fus_*H* correspond to typical values reported for form I of these APIs, as collected by Acree and Chickos in their compendia [28,29]. The *T*_g_ values obtained in this work for PAR and PRO are also close to those reported in the literature [13,30,31]. We note that the *T*_g_ values depend on the thermal history of the sample, as well as on the experimental conditions under which they are measured, which may lead to differences in these values in the order of units of °C.

### 2.2. Binary Solid–Liquid Phase Diagrams

**Figure 1:**
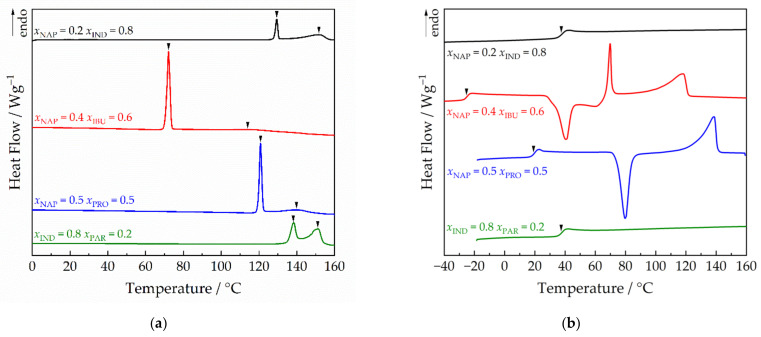
Examples of DSC thermograms. (**a**) Thermograms recorded for initial crystalline mixtures at a heating rate of 2 °C min^−1^. Arrows indicate eutectic and liquidus peaks. (**b**) Thermograms obtained after melting crystalline mixtures, their subsequent quenching, and heating by 10 °C min^−1^. Arrows indicate glass transition temperatures.

The thermograms obtained for the binary mixtures studied were typical of eutectic systems (Figure 1a). For all binary systems and most of the compositions studied, CAMs characterized by a single glass transition temperature (*T*_g_) were obtained by quenching the melt of initially crystalline binary physical mixtures during DSC analysis (Figure 1b). As NAP is a poor glass former, i.e., it has a high tendency to recrystallize, the CAMs were successfully formed only up to *x*_NAP_ = 0.7 in the mixture with IND and *x*_NAP_ = 0.5 in the mixtures with IBU and PRO. For mixtures exceeding the given NAP mole fraction, recrystallization appeared during the cooling of the melt, while for mixtures with a lower NAP content, recrystallization was observed on the heating curve at temperatures above *T*_g_ (e.g., NAP-IBU or NAP-PRO in Figure 1b) or no recrystallization from melt was detected during heating (e.g., NAP-IND in Figure 1b).

**Figure 2:**
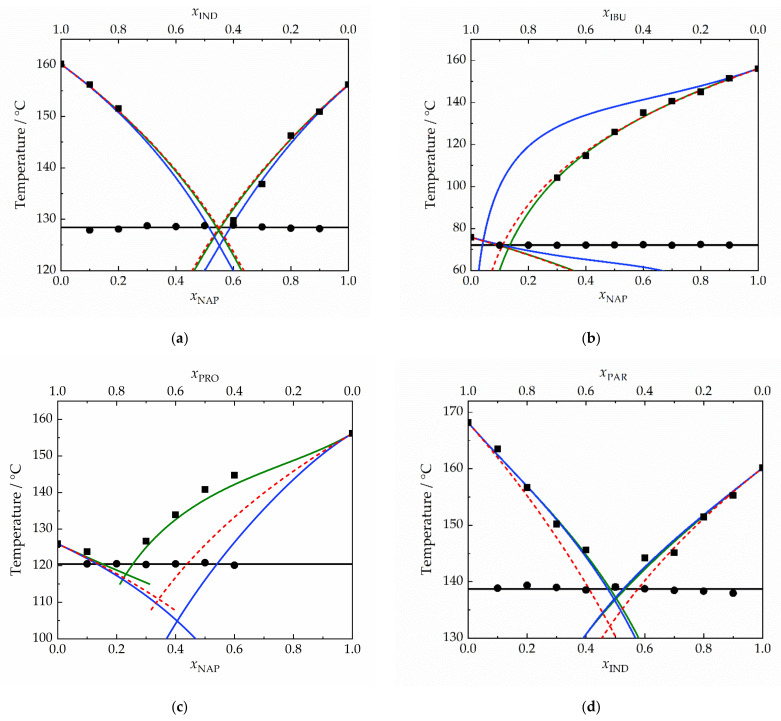
Phase diagrams for (**a**) NAP-IND, (**b**) NAP-IBU, (**c**) NAP-PRO, and (**d**) IND-PAR. Black squares: experimental liquidus temperatures *T*_L_; black circles: experimental eutectic temperatures *T*_E_; solid blue line: *T*_L_ predicted by PC-SAFT EOS (*k*_ij_ = 0); solid green line: *T*_L_ predicted by COSMO-RS-AMS; dashed red line: ideal solubility; black solid line: mean value of *T*_E_. The PC-SAFT calculations involving PRO are based on approximative parametrization (see Section 3.3.2).

The eutectic temperatures (*T*_E_) evaluated from thermograms as extrapolated onset temperatures showed a negligible variation with composition (in accordance with theory, see Figure 2) and the mean values are reported in Table 2. The standard deviation of the mean was significantly less than the uncertainty in the determination of phase transition temperatures. The experimentally obtained *T*_L_, evaluated as the top of the liquidus peak (as recommended by Höhne [9]), are listed in Table 3 and shown in Figure 2. The eutectic compositions (*x*_E_) were estimated based on Tammann plots [33] (see Figure S2 in the Supplementary Materials). Close to the eutectic composition, the liquidus peak overlapped with the eutectic peak, which did not allow us to evaluate *T*_L_ for these compositions and made the integration of eutectic peak, and thus the eutectic composition estimation using the Tammann plots, less reliable.

The experimental SLE data were used to evaluate the performance of two computational models, PC-SAFT EOS and COSMO-RS-AMS, to predict the phase diagrams for these systems. To calculate the solubility, i.e., the liquidus curves, two types of thermodynamic data are needed (see Equation (1)): (i) thermodynamic fusion properties of pure APIs and (ii) the activity coefficients ($\gamma_{\mathrm{API}}^{L}$) of a given API in the liquid solution. The required melting temperatures, fusion enthalpies, and differences in the liquid and crystalline heat capacities are listed in Table 1. To predict $\gamma_{\mathrm{API}}^{L}$, PC-SAFT EOS and COSMO-RS-AMS were employed. As the fusion thermodynamic data used in Equation (1) were determined experimentally (as is common because it is known that their prediction is burdened with large uncertainties and their determination is rather straightforward), the assessment of the performance of the two models reduces to a comparison of the quality of the $\gamma_{\mathrm{API}}^{L}$.prediction. To make this comparison as fair as possible, PC-SAFT EOS was applied with the binary interaction parameters *k*_ij_ set to 0 (for details, see Section 3.3.2), i.e., the $\gamma_{\mathrm{API}}^{L}$ prediction was carried out based on solely pure-component parameters for given APIs without any experimental input from the binary systems studied. COSMO-RS-AMS is a quantum-chemistry-based model that requires only the molecular structure as input for the $\gamma_{\mathrm{API}}^{L}$ prediction.

The liquidus curves calculated using the two computational models are plotted together with the experimental data in Figure 2. Ideal solubility calculations are also shown in the phase diagrams to assess the ability of the two models to predict the direction of deviations from ideality. As shown in Figure 2, the experimental liquidus curves in the binary systems NAP-IND (Figure 2a) and NAP-IBU (Figure 2b) are very close to those predicted based on the ideal solubility assumption, i.e., using $\gamma_{\mathrm{API}}^{L}$ equal to 1. For these two systems, COSMO-RS-AMS closely captures the observed trend in experimental *T*_L_ data, while PC-SAFT EOS performs well only for the NAP-IND system, for which the predicted behavior by PC-SAFT EOS is also close to the ideal system. For the NAP-IBU system, PC-SAFT EOS predicts significant positive deviations, i.e., $\gamma_{\mathrm{API}}^{L}\;>\;1$, which is in disagreement with the experimental observation. For the IND-PAR system (Figure 2d), the trend in the experimental *T*_L_ data is well described by both models. Based on the experimental *T*_L_ data for NAP-PRO (Figure 2c), the system exhibits significant positive deviations from ideality, which is remarkably well captured by COSMO-RS-AMS, while the PC-SAFT EOS predicts negative deviations from ideality. As discussed in Section 3.3.2, the PC-SAFT parameter set for PRO was obtained using an approximate procedure owing to the unavailability of experimental thermodynamic data for pure PRO, which may be the reason PC-SAFT EOS does not provide satisfactory results for systems containing PRO. This situation points to the substantial limits of PC-SAFT EOS, a relatively highly parametrized model, for the initial screening of suitable excipients for a given API during which a high number of pairs of API–excipients are considered, including newly proposed or developed APIs or excipients. In such situations, it is highly probable that suitable thermodynamic data for PC-SAFT parametrization will not be available for all considered or preselected materials, and one would have to opt for approximative parametrization procedures, as in the case of PRO, which may lead to unreliable phase diagram predictions and, subsequently, API–excipient compatibility ranking.

The comparison of *T*_E_ and *x*_E_ obtained as an intersection of liquidus curves calculated using the two models and using the assumption of ideal solubility with the experimentally determined values are provided in Table 2. As mentioned above, we consider *x*_E_ determined based on the Tammann plots to possess higher uncertainty owing to overlapping eutectic and liquidus peaks for compositions close to the eutectic composition, which makes the integration of eutectic peak less reliable. Table 3 presents the comparison between the calculated and experimental *T*_L_ values. On the basis of these quantitative results, it can be concluded that COSMO-RS-AMS provides overall more reliable predictions of SLE for the systems studied than PC-SAFT EOS. Given the fact that PC-SAFT EOS is a more parametrized model compared with COSMO-RS-AMS and its parametrization requires the thermodynamic data (e.g., solubility in organic solvents, liquid density, or vapor pressure) for each pure component forming the mixture, which may not be easily accessible, especially for newly developed APIs, COSMO-RS-AMS seems to be a more suitable model, allowing for rapid initial screening of suitable excipient candidates for an API under consideration, formulation composition, or processing conditions. To conclude, in addition to a superior performance in predicting the binary phase diagrams for the systems studied, the lower input data requirement of COSMO-RS-AMS (only optimized molecular structure must be provided) presents a significant advantage of this model compared with PC-SAFT EOS, especially when screening of the compatibility of a high number of API–excipient pairs is required.

The phase behavior of the NAP-IND system was previously studied by Rades and co-workers [2,10,11]. Phase diagrams are presented only in graphical form and modeled as ideal systems in [2,10]. In addition, the differences between the crystalline and liquid heat capacities were neglected in the solubility calculations. In [11], only the eutectic composition evaluated based on the dependence of the enthalpy of the eutectic peak on composition is reported (*x*_NAP_ = 0.60), which is in close agreement with the values obtained in this work (see Table 2). Experimentally determined *T*_E_ = 127.9 °C [10] agrees well with the value obtained in this work (*T*_E_ = 128.4 ± 0.3 °C), while a slightly higher *T*_E_ of about 130 °C can be read from the graphical representation of the phase diagram in [2]. The eutectic composition reported in [2] (*x*_NAP_ = 0.55) was obtained as an intersection of liquidus curves calculated based on the assumption of ideal solubility, while a slightly higher value (*x*_NAP_ = 0.60) was determined experimentally by the authors in [10]. However, it is not clear by which method this value was derived from the experimental data (the experimental data points seem to be only connected by connecting lines and not described by correlation or computational model). Despite certain small differences and unclear data treatment in [10], it can be concluded that the phase diagrams presented in [2,10] and the eutectic composition reported in [11] agree well with the results of this work.

For the IND-PAR system, Fael and Demirel [18] reported that the physical mixture exhibited a melting peak at 142 °C, which can be associated with the eutectic temperature. This value is slightly higher than that determined in this work (*T*_E_ = 138.7 °C).

### 2.3. Kinetic Stabilization of CAMs and Glass Transition of Binary Mixtures

As mentioned above, all initially crystalline physical mixtures were transformed to the amorphous state for compositions up to *x*_NAP_ = 0.5 for the NAP-IBU and NAP-PRO and *x*_NAP_ = 0.7 for the NAP-IND system. The mixture IND-PAR formed CAMs in the whole concentration interval. Although IBU, PRO, and IND have all been classified as good glass formers (Class 3 according to the classification proposed by Baird et al. [13]), IND offers the highest stabilization for NAP, an API with a high tendency to recrystallize (Class 1). It is also important to note that the mixtures close to the eutectic composition were successfully transformed into CAMs for all systems studied, which is a prerequisite step for the subsequent monitoring of their physical stability.

The glass transition temperature of binary mixtures plays an important role in the kinetic stabilization of CAMs. The physical stability of CAMs is typically proportional to the difference between its *T*_g_ and the storage temperature. The *T*_g_ values obtained for the CAMs studied are listed in Table S1 in the Supplementary Materials. Based on the physical stability study of three CAMs, including NAP-IND, Kissi et al. [10], and Beyer et al. [11], CAMs corresponding to the eutectic composition form the most stable CAMs. *T*_g_ values at *x*_E_ are close to typical storage temperature of 25 °C for NAP-IND (*T*_g_(*x*_E_) ≈ 23.8 °C) and NAP-PRO (*T*_g_(*x*_E_) ≈ 23.3 °C), significantly below for NAP-IBU (*T*_g_(*x*_E_) ≈ −40.7 °C, *x*_E_ was assumed to be *x*_NAP_ = 0.1), and slightly higher for IND-PAR (*T*_g_(*x*_E_) ≈ 30.7 °C). Although the kinetic stabilization of NAP-IND and IND-PAR CAMs possessing the eutectic composition derived on the basis of their *T*_g_ values is rather limited, Kissi et al. [10] found that CAMs did not show any sign of recrystallization in about 35 days (the range was 31 to 37 days) when stored at room temperature under dry conditions. The prolonged physical stability for NAP-IND CAMs was also reported by Löbmann et al. [2] (*x*_NAP_ = 0.5, storage temperatures of 4 and 25 °C, dry conditions, stability of at least 21 days) and Beyer et al. [11] (*x*_NAP_ = 0.6, storage temperature of 21 °C, dry conditions, monitoring period of 56 and 112 days). Fael and Demirel [18] reported that the physical stability of IND-PAR CAMs for 2:1, 1:1, and 1:2 molar ratios was up to 7 months (various storage conditions were examined: 4, 25, and 40 °C under dry conditions and 29 °C under mild humid conditions and relative humidity of 55%). The optimum composition in terms of physical stability was found to be a molar ratio of 2:1, followed by a molar ratio of 1:1. The literature findings summarized above, along with the *T*_g_ values of NAP-IND and IND-PAR CAM, which are close to typical storage temperatures, suggest that intermolecular interactions play a significant role in stabilizing CAMs.

It is important to mention that, in both studies [10,11] indicating the blends corresponding to eutectic composition as the most stable CAM, the eutectic composition was close to that of the equimolar mixture. Therefore, as stated by Kissi [10], the observed relation between CAM physical stability and the eutectic composition should be investigated for mixtures whose eutectic composition is located further away from the equimolar mixture. Such a system can be, e.g., NAP-PRO, whose phase behavior was studied in this work (it forms CAM at eutectic composition, which significantly differs from the equimolar composition).

**Figure 3:**
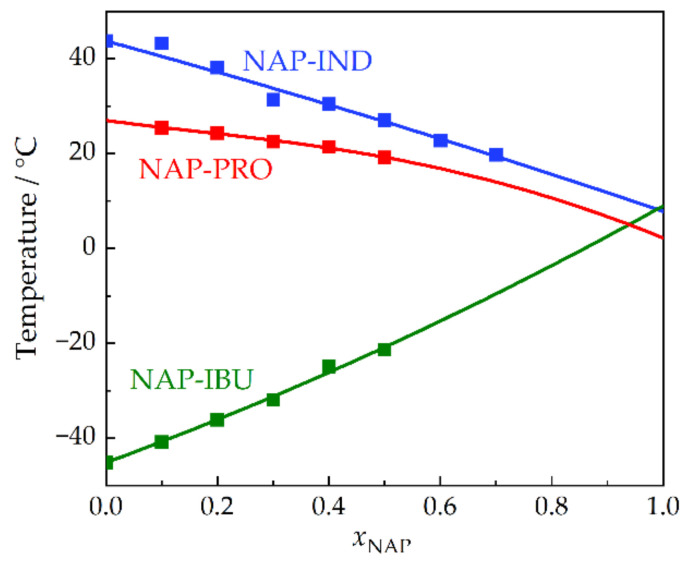
*T*_g_ values for mixtures NAP-IND, NAP-IBU, and NAP-PRO measured by DSC (represented by squares) fitted with the Kwei equation, Equation (8) (represented by lines), as a function of molar fraction of NAP, *x*_NAP_. The fit by the Gordon–Taylor equation, Equation (7), is not shown as it cannot be distinguished from that by the Kwei equation.

Significant discrepancies in the *T*_g_ values for NAP were identified in the literature [2,14,15,16]. As reliable *T*_g_ values for pure components (APIs and excipients) that form amorphous formulations represent key information in the evaluation of their kinetic stability, we attempted to clarify the situation regarding the *T*_g_ of pure NAP in this work based on measuring and modeling *T*_g_ of binary amorphous mixtures in which NAP was stabilized in the amorphous state. The experimental values on *T*_g_ of the three mixtures containing NAP, i.e., NAP-IND, NAP-IBU, and NAP-PRO, listed in Table S1 in the Supplementary Materials, were correlated by the Gordon–Taylor equation, Equation (7), and the Kwei equation, Equation (8), with *T*_g_ of pure NAP (*T*_g, NAP_) as a fitted parameter. The modeled *T*_g_ curves are shown in Figure 3 and the obtained *T*_g, NAP_ values are summarized in Table 4. The mean value of *T*_g, NAP_ = 6.4 ± 1.4 °C (the uncertainty quoted is the standard deviation of the mean) is in excellent agreement with the values determined by Paudel et al. [14] (*T*_g, NAP_ = 6.2 °C, amorphous NAP prepared by spray drying) and by Löbmann et al. [2] (*T*_g, NAP_ = 5.0 °C, amorphous NAP prepared melt quenching), but differs significantly from the value reported by Blaabjerg et al. [15] (*T*_g, NAP_ = 56.1 °C, sample prepared by melt quenching method) and adopted, for example, by Kawakami [16]. Some variation in the measured *T*_g_ values is expected because of their dependence on the thermal history of the amorphous material and, in general, on the experimental conditions using which they are determined. However, such a large deviation of approximately 50 °C cannot be explained by these phenomena and the value reported by Blaabjerg et al. [15] can be considered erroneous. In this work, we provide clear evidence that *T*_g, NAP_ is about 6 °C, in accordance with the two previous studies [2,14].

## 3. Materials and Methods

### 3.1. Samples Description

The APIs studied in this research are listed with their basic characteristics in Table 5. APIs were used as received from the manufacturer without further purification. The thermodynamic fusion properties and *T*_g_ values determined in this work are provided in Table 1.

### 3.2. Experimental Methods

#### 3.2.1. Differential Scanning Calorimetry

A Q1000 differential scanning calorimeter (TA Instruments, Inc., New Castle, DE, USA) was used to determine melting temperatures and enthalpies of pure APIs, phase diagrams, and *T*_g_ values. The physical mixtures containing two different APIs in different molar ratios were prepared by grinding with a mortar and pestle for 10 min. Approximately 5–10 mg of sample was then hermetically sealed in aluminum pans and analyzed by DSC. The experiments consisted of two heating cycles. The heating rate of 2 °C min^−1^ was applied for SLE measurements during the first heating run. Subsequently, the melt was quenched using a cooling rate of 10 °C min^−1^ and *T*_g_ values were measured during the second heating cycle with a heating rate of 10 °C min^−1^.

#### 3.2.2. X-ray Powder Diffraction

X-ray powder diffraction analysis was performed to identify the polymorphic forms of the APIs studied using a 𝜃–λ powder diffractometer X′Pert3Powder in Bragg–Brentano parafocussing geometry using wavelength CuK radiation (𝜆 = 1.5418 Å, *U* = 40 kV, *I* = 30 mA). Data were gathered using an ultrafast detector 1D PIXcel angular range 5–50° (2𝜃) with a step size of 0.039° (2𝜃) and 0.7 s for each step. HighScorePlus 4.0 software was used to analyze the obtained diffractograms. The polymorphic forms were identified based on the comparison to the CSD [26].

### 3.3. Computational Methods

#### 3.3.1. Modeling of Solid–Liquid Equilibria

The API solubility (mole fraction $x_{\mathrm{API}}^{L}$) was calculated according to the following equation:

(1) $$x_{\mathrm{API}}^{L}=\frac{1}{\gamma_{\mathrm{API}}^{L}}\exp\left[-\;\frac{\Delta_{\mathrm{fus}}H}{RT}\left(1\;-\;\frac{T}{T_{m}}\right)\;-\;\frac{1}{RT}\int_{T_{m}}^{T}\Delta_{\mathrm{fus}}C_{p}dT+\frac{1}{R}\int_{T_{m}}^{T}\frac{\Delta_{\mathrm{fus}}C_{p}}{T}dT\right],$$

where $\Delta_{\mathrm{fus}}H$ is the fusion enthalpy of pure API, *T*_m_ is its corresponding melting temperature (in K), *T* is absolute temperature (in K), $\Delta_{\mathrm{fus}}C_{p}$ is the difference between the isobaric heat capacity of the liquid and the crystalline phase, and *R* is the universal gas constant. $\gamma_{\mathrm{API}}^{L}$ is the activity coefficient of one of the APIs in the liquid API–API mixture. $\gamma_{\mathrm{API}}^{L}$ was calculated using the PC-SAFT equation of state, COSMO-RS-AMS, or set as 1 in the case of ideal solubility calculations.

#### 3.3.2. PC-SAFT Equation of State

According to the PC-SAFT equation of state, the residual Helmholtz energy (*a*^res^) is commonly calculated as a sum of three different contributions resulting from repulsion (hard chain), van der Waals attraction (dispersion), and hydrogen bonding (association) [19,20]:

(2) $$a^{\mathrm{res}}{=a}^{\mathrm{hc}}{+a}^{\mathrm{disp}}{+a}^{\mathrm{assoc}},$$

From Equation (2), other thermodynamic properties of a system can be calculated, including the activity coefficient $\gamma_{\mathrm{API}}^{L}$ [19]. PC-SAFT considers molecules to be chains constituted by spherical segments. Materials are then characterized using the following set of pure component parameters: the number of segments within a chain (*m*_i_), the diameter of the segment (*σ*_i_), the dispersion energy parameter (*ε*_i_/*k*, *k* is the Boltzmann constant), the association energy parameter (*ε*_i_^assoc^/*k*), the association volume (*κ*_i_^assoc^), and the number and type of association sites per molecule (*N*_i_^assoc^). Given by the semi-empirical nature of the model, values of these parameters are routinely fitted to experimental data. Values of the PC-SAFT parameters for the studied APIs are given in Table 6. The parameters for IND, IBU, NAP, and PAR were taken from the literature [34]. For each API, they were obtained by fitting them to the properties of the pure liquid API along with the API solubility data in a row of pure solvents. However, the PC-SAFT parameters for PRO were not found in the available literature. At the same time, the literature lacked experimental solubility data of PRO in pure solvents. Therefore, an alternative parametrization approach was applied to PRO. First, the parameter *κ*_i_^assoc^ was set to a constant value of 0.01, as usual for APIs in the literature [34]. For the association energy parameter, *ε*_i_^assoc^/*k*, we used the value typical for the phenolic OH group (1650 K) [35,36], while the structural parameters *m*_i_ and σ_i_ were estimated using a group contribution approach [37,38]. Finally, the dispersion energy parameter, *ε*_i_/*k*, remained the only adjustable parameter and was fitted to the only experimental solubility data point of PRO in a pure solvent (ethanol) available in the literature reported by Yagi et al. [39] (for completeness, the PC-SAFT parameters for ethanol were taken from [20]). Therefore, owing to this crudely approximative nature of the PC-SAFT parameter set for PRO, the PC-SAFT results for systems with this API should be taken with caution and considered to be only an illustration of what to expect from the model when parametrized in an alternative way because of the inaccessibility of experimental data. To emphasize this, all results from PC-SAFT for PRO are denoted with “!”.

For the calculation of the thermodynamic properties of mixtures, the combination rules for the cross parameters *σ*_ij_ and *ε*_ij_*/k* between components i and j are applied as follows [19]:

(3) $$\sigma_{\mathrm{ij}}=\frac{1}{2}\left(\sigma_{i}{+\sigma }_{j}\right),$$

(4) $$\varepsilon_{\mathrm{ij}}=\sqrt{\varepsilon_{i}\varepsilon_{j}}\left({1\;-\;k}_{\mathrm{ij}}\right),$$

where *k*_ij_ is the binary interaction parameter, which can be calculated by fitting the experimental solubility data or set to 0. In the case of *k*_ij_ = 0, SLE data are calculated based only on the PC-SAFT parameters of the pure components. In this work, the PC-SAFT was used solely with *k*_ij_ = 0, i.e., no experimental data for the binary systems studied were employed. Combination rules for the association parameters can be found elsewhere [20].

$\gamma_{\mathrm{API}}^{L}$ is determined from the following equation:

(5) $${\ln\gamma }_{i}^{L}{=\ln\varphi }_{i}^{L}{\;-\;\ln\varphi }_{0,i}^{L},$$

where $\varphi_{i}^{L}$ is the fugacity coefficient of API in the liquid API–API mixture and $\varphi_{0,i}^{L}$ is the fugacity coefficient of the pure liquid. The fugacity coefficients are obtained from the general equation:

(6) $${\ln\varphi }_{i}^{L}{=a}^{\mathrm{res}}+{\left(\frac{\partial a^{\mathrm{res}}}{\partial x_{i}}\right)}_{{T,r,x}_{k\neq i}}-\underset{j}{\sum }x_{j}\left[{\left(\frac{\partial a^{\mathrm{res}}}{\partial x_{j}}\right)}_{{T,r,x}_{k\neq j}}\right]+Z\;-\;1\;-\;\mathrm{lnZ}$$

where *a*^res^ is the reduced residual Helmholtz energy obtained from PC-SAFT, *Z* is the compressibility factor, and *ρ* is the system molar density.

#### 3.3.3. COSMO-RS-AMS

COSMO-RS represents an efficient and successfully used methodology for predicting the thermodynamic properties of fluid systems. It combines quantum chemical calculations of molecular properties and a statistical mechanical procedure to obtain the macroscopic properties of a solution [21]. The quantity that bridges these two steps is the sigma-profile of a molecule, which is a surface histogram with respect to the charge density calculated quantum chemically using density functional theory (DFT) and the COSMO model to imitate the solvent environment. As such, the COSMO-RS methodology allows for a priori predicting of phase equilibria without any experimental data, also including SLE in pharmaceutical systems (e.g., [40,41]). Multiple implementations of the COSMO-RS methodology are available in the literature (e.g., [22,42,43]). In this work, we used COSMO-RS as implemented in the Amsterdam Modeling Suite (AMS), version 2022.101 [22,44] (COSMO-RS-AMS). The sigma-profiles for IND, IBU, NAP, and PAR used in this work were taken from Klajmon [41], while that for PRO was determined in this work using AMS. Molecular geometry of PRO, which is the only input in producing the sigma-profile, was taken from the HAXHET01 crystal structure of form I [45] available in the CSD [26], which was considered to be appropriately representing predominant molecular geometries of PRO in the condensed phase [41].

#### 3.3.4. Modelling of the Glass Transition Temperature Curve

The Gordon–Taylor equation [46] and the Kwei equation [47] were used to model *T*_g_ values of the binary mixtures studied and to estimate the *T*_g_ value for pure NAP. The Gordon–Taylor equation is defined as follows [46]:

(7) $$T_{g}=\frac{x_{1}T_{g1}{+\mathrm{kx}}_{2}T_{g2}}{x_{1}{+\mathrm{kx}}_{2}},$$

where *T*_g_ is the glass transition temperature of a binary mixture, *T*_g1_ and *T*_g2_ are the glass transition temperatures of pure APIs, and *x*_1_ and *x*_2_ are their molar fractions. *k* is a parameter that is determined by fitting experimentally measured *T*_g_ values. By adding the second fitting parameter *q* to the Gordon–Taylor equation, Equation (7), the Kwei equation is obtained, defined as follows [47]:

(8) $$T_{g}=\frac{x_{1}T_{g1}{+\mathrm{kx}}_{2}T_{g2}}{x_{1}{+\mathrm{kx}}_{2}}{+\mathrm{qx}}_{1}x_{2},$$

where *k* has the same meaning as in the Gordon–Taylor equation, Equation (7), and *q* is the second fitted parameter.

## 4. Conclusions

In this work, the performance of the two computational models, PC-SAFT EOS and COSMO-RS-AMS, to predict the phase diagrams for four binary systems combining two APIs was evaluated based on the comparison to experimental SLE data. Overall, COSMO-RS-AMS outperformed PC-SAFT EOS and, given the fact that it is a significantly less parametrized model compared with PC-SAFT EOS, it can be considered as a more suitable computational tool for initial screening of phase diagrams of API–excipient pairs, and thus their compatibility.

Melting of the initial binary crystal mixtures and their subsequent quenching lead to the formation of CAMs for all binary systems and most of the studied compositions. NAP, an API with a high tendency to recrystallize, was successfully stabilized in its amorphous form in mixtures with IND, IBU, and PRO (APIs with good glass-forming ability), which allowed us to determine its *T*_g_ and reconcile the values published in the literature.

## Figures and Tables

**Table 1:** Thermodynamic fusion properties and glass transition temperatures for the APIs studied.

Compound	Polymorph ^a^	*T*_m_/°C ^b^	$\Delta_{\mathrm{fus}}H/\mathrm{kJ}\;{\mathrm{mol}}^{-1\;b}$	$\Delta_{\mathrm{fus}}C_{p}/J\;K^{-1}\;{\mathrm{mol}}^{-1}$	*T*_g_/°C ^b^
indomethacin	form γ (INDMET)	160.2	38.1	117.5 ^c^	42.3
(RS)-ibuprofen	form I (IBPRAC)	75.8	26.4	55.8 ^c^	−43.8
(S)-naproxen	form I (COYRUD11)	156.0	32.4	99.3 ^c^	6.4 ^d^
paracetamol	form I (HXACAN34)	168.2	27.1	99.8 ^e^	25.7
probucol	form I (HAXHET01)	126.0	35.3	124.7 ^f^	22.0

^a^ Commonly used name for a given polymorph. The code in the brackets is the polymorph identifier in the CSD. ^b^ This work. The combined expanded uncertainty *U*_c_ (0.95 level of confidence) in the determination of *T*_m_ values, *T*_g_ values, and $\Delta_{\mathrm{fus}}H$ values is estimated to be 0.3 °C and 3%, respectively. ^c^ Values taken from Štejfa et al. [27]. ^d^ This work. The value was obtained by extrapolation from the measured *T*_g_ values of binary mixtures with NAP (see Section 3.3.4). ^e^ The value was determined based on the isobaric heat capacity data reported by Neau et al. [32]. ^f^ The value was determined in our laboratory by a combination of Tian-Calvet, power-compensated DSC, and relaxation calorimetry.

**Table 2:** Measured and calculated eutectic temperatures *T*_E_ and obtained eutectic compositions *x*_E_ (mole fraction).

System	*T*_E_ (*x*_E_)/°C ^c^				*x*_E_^b^
	Experiment ^a^	Ideal Solubility	PC-SAFT (*k*_ij_ = 0)	COSMO-RS-AMS	Tammann Plot
NAP (1)–IND (2)	128.4	128.1 (*x*_1_ = 0.55)	124.9 (*x*_1_ = 0.55)	127.8 (*x*_1_ = 0.55)	*x*_1_ = 0.58
NAP (1)–IBU (2)	72.1	71.4 (*x*_1_ = 0.11)	74.2 (*x*_1_ = 0.04)	70.5 (*x*_1_ = 0.13)	-
NAP (1)–PRO (2)	120.5	110.8 (*x*_1_ = 0.34)	104.9 (*x*_1_ = 0.41) (!) ^d^	117.7 (*x*_1_ = 0.23)	*x*_1_ = 0.33
IND (1)–PAR (2)	138.7	132.2 (*x*_1_ = 0.48)	136.7 (*x*_1_ = 0.50)	137.0 (*x*_1_ = 0.50)	*x*_1_ = 0.40

^a^ The combined expanded uncertainty *U*_c_ (0.95 level of confidence) in the determination of *T*_E_ values is estimated to be 0.3 °C. ^b^ The combined expanded uncertainty *U*_c_ (0.95 level of confidence) in the determination of *x*_E_ values using the Tammann plots [33] was estimated to be 0.05. The uncertainty estimation was made based on the uncertainties associated with the determination of enthalpies of the eutectic peak Δ*H*_E_. ^c^ Calculated as an intersection of liquidus curves. ^d^ The results may be affected by the approximative set of PC-SAFT parameters for PRO (for details, see Section 3.2.2).

**Table 3:** Experimental liquidus temperatures ***T*_L_** and their comparison to the calculated values.

	NAP (1)–IND (2)				NAP (1)–IBU (2)			
*x*_1_	Experiment *T*_L_/°C ^a^	Δ*T/*°C ^b^			Experiment *T*_L_/°C ^a^	Δ*T/*°C ^b^		
		PC-SAFT	COSMO-RS-AMS	Ideal Solubility		PC-SAFT	COSMO-RS-AMS	Ideal Solubility
0.1	156.2	−0.4	−0.2	−0.3	-	-	-	-
0.2	151.6	−0.8	0.1	−0.5	-	-	-	-
0.3	-	-	-	-	104.1	24.2	−0.1	1.7
0.4	-	-	-	-	114.6	19.4	1.3	2.1
0.5	-	-	-	-	125.9	12.1	−0.6	−0.3
0.6	129.8	−0.4	2.3	2.7	135.1	6.2	−1.9	−2.0
0.7	136.8	0.8	2.4	2.7	140.5	4.0	−0.6	−0.7
0.8	146.3	−1.5	−0.7	−0.6	145.0	2.9	0.9	0.7
0.9	150.9	0.0	0.3	0.3	151.4	0.3	−0.2	−0.3
σ ^c^	-	0.6	1.0	1.2	-	9.9	0.8	1.1
	**NAP (1)–PRO (2)**				**IND (1)–PAR (2)**			
***x*_1_**	**Experiment *T*_L_/°C ^a^**	**Δ*T/*°C ^b^**			**Experiment *T*_L_/°C ^a^**	**Δ*T/*°C ^b^**		
		**PC-SAFT (!) ^d^**	**COSMO-RS-AMS**	**Ideal** **Solubility**		**PC-SAFT**	**COSMO-RS-AMS**	**Ideal** **Solubility**
0.1	123.8	−1.9	−1.5	−1.7	168.2	−1.1	−1.0	−1.5
0.2	-	-	-	-	163.5	0.3	0.3	−1.4
0.3	126.7	−14.9	−1.7	−13.7	156.7	0.8	1.0	−2.4
0.4	133.9	−28.6	−1.3	−17.1	150.2	−2.0	−0.8	−6.0
0.5	140.8	−24.7	−2.8	−15.1	145.6	−2.6	−2.2	−5.6
0.6	144.7	−18.3	−2.5	−11.4	139.1	−1.6	−2.0	−4.0
0.7	-	-	-	-	144.2	2.3	2.0	0.9
0.8	-	-	-	-	145.1	0.4	0.2	−0.2
0.9	-	-	-	-	151.5	0.8	0.8	0.6
σ ^c^	-	14.7	1.6	9.8	-	1.3	1.1	2.5

^a^ The combined expanded uncertainty *U*_c_ (0.95 level of confidence) in the determination of *T*_L_ values is estimated to be 0.3 °C. ^b^ Deviation between the calculated and the experimentally determined liquidus temperature, ${\Delta T=T}_{L}-T_{L}^{\exp}$. ^c^ Average absolute deviation calculated as $\sigma =\frac{1}{N}\overset{N}{\underset{i=1}{\sum }}\left|T_{L}-T_{L}^{\exp}\right|$. ^d^ The results may be affected by the approximative set of PC-SAFT parameters for PRO (for details, see Section 3.2.2).

**Table 4:** Parameters of the Gordon–Taylor equation, Equation (7); Kwei equation, Equation (8); and obtained *T*_g_ of pure NAP (*T*_g, NAP_).

System	Gordon–Taylor Equation		Kwei Equation		
	*k*	*T*_g, NAP_/°C	*k*	*q*	*T*_g, NAP_/°C
NAP-IND	0.90	7.9	1.00	3.80	7.8
NAP-IBU	0.79	9.8	1.00	−10.95	9.0
NAP-PRO	0.42	1.7	2.14	36.47	2.2
IND-PAR	1.80	-	3.08	8.54	-

**Table 5:** Description of APIs studied.

Compound	CAS RN	Abbreviation	Supplier	Mole Fraction Purity ^a^
indomethacin	53-86-1	IND	Merck	0.998
(RS)-ibuprofen	15687-27-1	IBU	Zentiva	0.998
(S)-naproxen	22204-53-1	NAP	Merck	0.999
paracetamol	103-90-2	PAR	Merck	0.999
probucol	23288-49-5	PRO	Merck	0.997

^a^ Purity determined using DSC and the van’t Hoff equation according to the ASTM E928.

**Table 6:** PC-SAFT parameters of APIs studied.

Compound	*m*_i_	*σ*_i_/Å	*ε*_i_/*k*/K	$\varepsilon_{i}^{\mathrm{assoc}}/k/K$	$\kappa_{i}^{\mathrm{assoc}}$	$N_{i}^{\mathrm{assoc}}$
IND ^a^	7.8970	3.8225	374.51	1295.43	0.01135	6 (3/3)
IBU ^a^	5.4386	4.0179	309.40	516.469	0.08946	4 (2/2)
NAP ^a^	4.4122	4.1142	470.92	1202.65	0.00952	4 (2/2)
PAR ^a^	3.2357	3.9819	432.09	1635.92	0.05432	4 (2/2)
PRO ^b^	11.8500	3.8500	175.62	1650.00	0.01000	4 (2/2)

^a^ Values taken from Klajmon [34]. ^b^ Values determined in this work using an alternative approach that combined fitting, group contribution method, and structural similarity (see the text for details).

## Data Availability

All data relevant to this publication are included.

## Supplementary Materials

The following supporting information can be downloaded at https://www.mdpi.com/article/10.3390/molecules28062492/s1. Figure S1: XRPD patterns of APIs studied; Figure S2: Tammann plots for the systems studied; Table S1: *T*_g_ values obtained for binary systems studied.
