# Supplementary material for: Solid–Liquid Equilibrium in Co-Amorphous Systems: Experiment and Prediction
Source: Molecules. 2023 Mar 8;28(6):2492. doi: 10.3390/molecules28062492 (PMC10052153; doi:10.3390/molecules28062492)
Supplement: Supplementary file 1 [file molecules-28-02492-s001.zip › molecules-2268632-supplementary.pdf]

Table S1. T_g values obtained for binary systems studied.

x_1	T_g / °C			
	**NAP (1) – IND (2)**	**NAP (1) – IBU (2)**	**NAP (1) – PRO (2)**	**IND (1) – PAR (2)**
0.1	43.2	−41.1	25.6	24.2
0.2	37.3	−35.9	24.4	27.5
0.3	31.3	−31.1	22.5	28.8
0.4	30.4	−25.0	21.4	31.8
0.5	27.0	−21.4	19.2	33.5
0.6	22.7	-	-	35.0
0.7	19.8	-	-	31.2
0.8	-	-	-	37.3
0.9	-	-	-	38.8

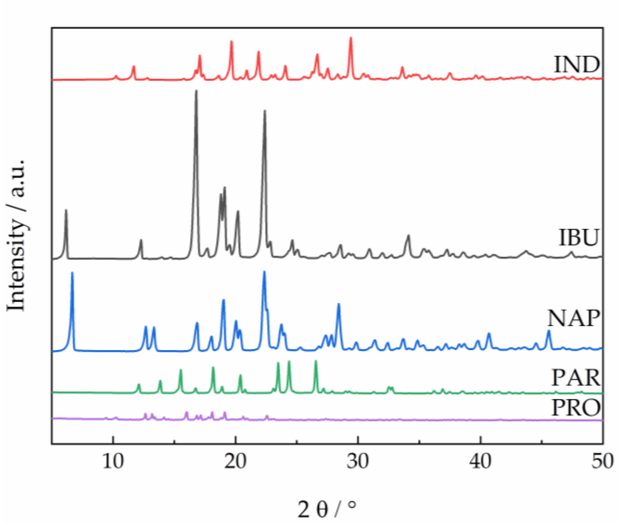

Figure S1. XRPD patterns of APIs studied. Based on comparison of diffractogams with the Cambridge structural database the following crystal structures were indentified. IND: form γ (INDMET), IBU: form I (IBPRAC), NAP: form I (COYRUD11), PAR: form I (HXACAN34), and PRO: form I (HAXHET01).

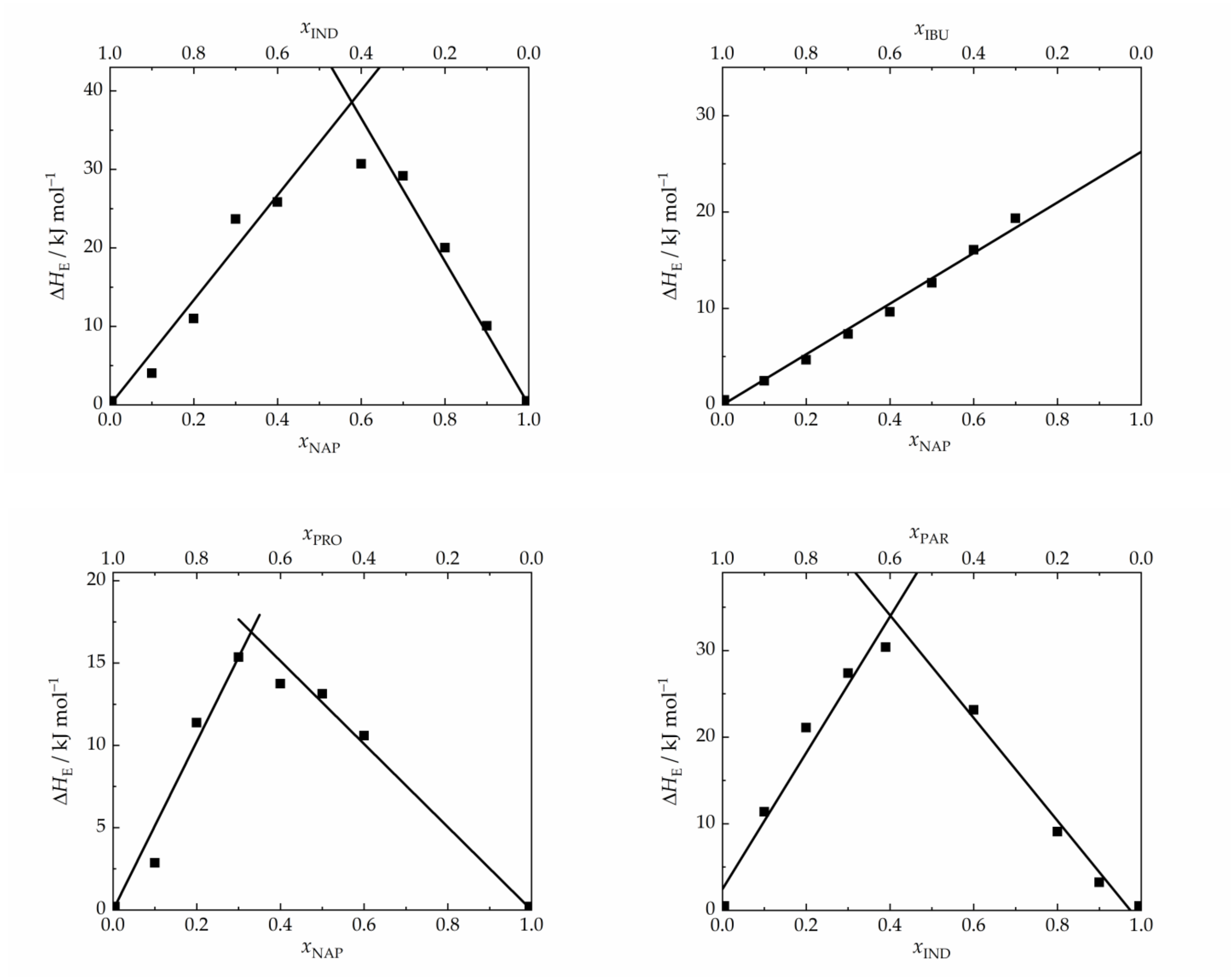

Figure S2. Tammann plots of studied systems. ΔH_E represents the enthalpy obtained by integration of eutectic peak.
